# A retrograde adeno-associated virus for collecting ribosome-bound mRNA from anatomically defined projection neurons

**DOI:** 10.3389/fnmol.2015.00056

**Published:** 2015-09-24

**Authors:** Denise R. Cook-Snyder, Alexander Jones, Leon G. Reijmers

**Affiliations:** ^1^Department of Neuroscience, School of Medicine, Tufts UniversityBoston, MA, USA; ^2^Department of Biology and Neuroscience Program, Carthage CollegeKenosha, WI, USA; ^3^Graduate Program in Neuroscience, Sackler School of Graduate Biomedical Sciences, Tufts UniversityBoston, MA, USA

**Keywords:** AAV9, Camk2a, EGFP-L10a, TRAP, projection neuron

## Abstract

The brain contains a large variety of projection neurons with different functional properties. The functional properties of projection neurons arise from their connectivity with other neurons and their molecular composition. We describe a novel tool for obtaining the gene expression profiles of projection neurons that are anatomically defined by the location of their soma and axon terminals. Our tool utilizes adeno-associated virus serotype 9 (AAV9), which we found to retrogradely transduce projection neurons after injection at the site of the axon terminals. We used AAV9 to express Enhanced Green Fluorescent Protein (EGFP)-tagged ribosomal protein L10a (EGFP-L10a), which enables the immunoprecipitation of EGFP-tagged ribosomes and associated mRNA with a method known as Translating Ribosome Affinity Purification (TRAP). To achieve high expression of the EGFP-L10a protein in projection neurons, we placed its expression under control of a 1.3 kb alpha-calcium/calmodulin-dependent protein kinase II (Camk2a) promoter. We injected the AAV9-Camk2a-TRAP virus in either the hippocampus or the bed nucleus of the stria terminalis (BNST) of the mouse brain. In both brain regions the 1.3 kb Camk2a promoter did not confer complete cell-type specificity around the site of injection, as EGFP-L10a expression was observed in Camk2a-expressing neurons as well as in neuronal and non-neuronal cells that did not express Camk2a. In contrast, cell-type specific expression was observed in Camk2a-positive projection neurons that were retrogradely transduced by AAV9-Camk2a-TRAP. Injection of AAV9-Camk2a-TRAP into the BNST enabled the use of TRAP to collect ribosome-bound mRNA from basal amygdala projection neurons that innervate the BNST. AAV9-Camk2a-TRAP provides a single-virus system that can be used for the molecular profiling of anatomically defined projection neurons in mice and other mammalian model organisms. In addition, AAV9-Camk2a-TRAP may enable the discovery of protein synthesis events that support information storage in projection neurons.

## Introduction

Projection neurons are responsible for the transmission of information between brain regions, and thereby play a central role in many physiological and pathophysiological processes in the brain. The classification of projection neurons is traditionally based on the location of their soma and their axon terminals. In addition to their anatomical heterogeneity, projection neurons are also heterogeneous at the molecular level (Arlotta et al., 2005; Sugino et al., 2006; Doyle et al., 2008; Heiman et al., 2008; Zeisel et al., 2015). A deeper understanding of this molecular heterogeneity can elucidate mechanisms by which projection neurons process and store information, and identify therapeutic targets for brain disorders that impact projection neurons (for example Shepherd, 2013). Achieving this will require improved methods that enable efficient molecular profiling of the large variety of projection neurons in the brain.

Different classes of projection neurons that project to different brain regions can be intermingled at the level of their soma. In addition, projection neurons are usually intermingled with other cell types. This complex cellular environment makes it challenging to collect biomolecules (for example DNA, RNA, or proteins) from a single class of projection neurons. Three types of methods are currently used to address this challenge (Okaty et al., 2011). First, samples from single projection neurons can be collected by aspiration through a micro-pipette or by laser-capture microdissection (Van Gelder et al., 1990; Lambolez et al., 1992; Kamme et al., 2003). Second, projection neurons from a single defined class can be collected by dissociating cells from brain tissue followed by sorting using a fluorescent reporter protein or a surface protein that is exclusively expressed within that class of projection neurons (Arlotta et al., 2005; Sugino et al., 2006; Dugas et al., 2008). Third, an epitope-tag can be exclusively expressed within a single class of projection neurons. By attaching the epitope-tag to a protein that binds to a group of biomolecules, for example a RNA-binding protein, immunoprecipitation can be used to collect these biomolecules from homogenized brain tissue (Doyle et al., 2008; Heiman et al., 2008; Sanz et al., 2009; Ekstrand et al., 2014).

Here, we present a novel viral tool for the molecular profiling of projection neurons that are anatomically defined by the location of their soma and axon terminals. Specifically, we developed a retrograde virus that expresses an epitope-tagged ribosomal protein. Injection of this virus at the site of the axon terminals results in expression of epitope-tagged ribosomes in retrogradely transduced projection neurons. After dissecting and homogenizing the brain region that contains the soma of these retrogradely transduced projection neurons, the epitope-tagged ribosomal proteins and associated mRNA can be collected using the Translating Ribosome Affinity Purification (TRAP) method (Heiman et al., 2008). We show that our viral tool can target various types of projection neurons with different locations of soma and axon terminals, and demonstrate its utility by collecting mRNA from basal amygdala projection neurons that innervate the bed nucleus of the stria terminalis (BNST).

## Materials and Methods

### Animals

All animal procedures were performed in accordance with the National Institutes of Health Guide for the Care and Use of Laboratory Animals and were approved by the Tufts University Institutional Animal Care and Use Committee. Wild type male C57BL/6J (JAX Stock Number 000664) and Friend Virus B-type (FVB; JAX Stock Number 004828) mice were given food and water *ad libitum* and were housed three to five animals per cage until the start of the experiment (8 weeks of age or older). Mice were kept on a regular light-dark cycle, and all experimental manipulations were done during the light phase. For this study a total of 10 mice were used in three experiments. Experiment 1, intrahippocampal adeno-associated virus (AAV) injection followed by immunohistochemistry: 3 C57BL/6J mice and 1 FVB mouse. Experiment 2, intra-BNST AAV injection followed by immunohistochemistry: 3 C57BL/6J mice. Experiment 3, intra-BNST AAV injection followed by TRAP: 3 C57BL/6J mice.

### Virus Production

**Figure 1:**
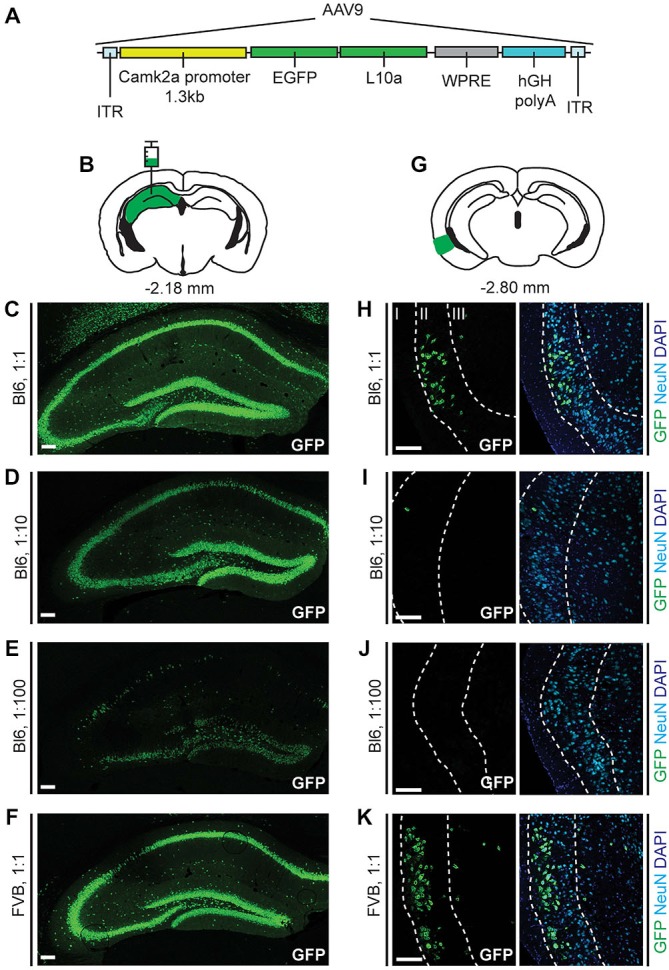
**Enhanced Green Fluorescent Protein (EGFP)-L10a expression in the hippocampus and entorhinal cortex after injection of adeno-associated virus serotype 9 (AAV9)-alpha-calcium/calmodulin-dependent protein kinase II (Camk2a)-Translating Ribosome Affinity Purification (TRAP) in the hippocampus. (A)** The AAV9-Camk2a-TRAP virus contains a 1.3 kb Camk2a promoter that drives expression of EGFP-tagged ribosomal protein L10a. ITR, Inverted terminal repeat; WPRE, woodchuck hepatitis virus posttranscriptional regulatory element; hGH polyA, human growth hormone polyA sequence. **(B)** AAV9-Camk2a-TRAP was injected into the hippocampus (bregma: −2.18 mm, hippocampus highlighted in green; coronal diagram modified from Paxinos and Franklin, 2001). **(C–F)** Injections were done in wild type C57BL/6J (Bl6) **(C–E)** or Friend Virus B-type (FVB) **(F)** mice using three different dilutions of the virus (1:1, 2.18 × 10^13^ viral genomes/ml; 1:10, 2.18 × 10^12^ viral genomes/ml; 1:100, 2.18 × 10^11^ viral genomes/ml). Immunohistochemistry was performed to label EGFP-L10a in the hippocampus (green). **(G)** Following AAV9-Camk2a-TRAP injection in the hippocampus, EGFP-L10a expression was also observed in the entorhinal cortex (bregma: −2.80 mm, entorhinal cortex highlighted in green). **(H–K)** Layers in the entorhinal cortex were delineated using the neuronal marker NeuN (cyan) and the nuclear marker 4′,6-diamidino-2-phenylindole (DAPI) (blue) (layers are labeled with roman numerals). Each entorhinal cortex image shown was obtained from the same brain as the hippocampal image on its left side. **(C–F,H–K)** Scale bars 100 μm.

Construct cloning and packaging of adeno-associated virus serotype 9 (AAV9)-alpha-calcium/calmodulin-dependent protein kinase II (Camk2a)-TRAP was performed by Virovek. The Enhanced Green Fluorescent Protein (EGFP)-L10a fusion protein coding sequence from pLD53.SC.EGFP-L10a (gift from Nathaniel Heintz, Rockefeller University) was cloned into a plasmid containing a 1.3 kb fragment of the mouse Camk2a promoter (Dittgen et al., 2004) to create a Camk2a-TRAP construct (Figure 1A). The construct was packaged into an AAV vector containing AAV2 inverted terminal repeats (ITRs) and AAV9 capsid protein (AAV9-Camk2a-TRAP). Viral titer was 2.18 × 10^13^ viral genomes/ml. The Camk2a promoter sequence in AAV9-Camk2a-TRAP was verified using an ABI 3130XL Automated DNA Sequencer (Life Technologies) at the Tufts University Core Facility.

### Stereotaxic Surgery

Mice were anesthetized with isoflurane, held in a stereotaxic apparatus (Kopf), and bilaterally injected with AAV9-Camk2a-TRAP in the hippocampus (1000 nl; AP −1.45 mm, ML ± 1.6 mm, DV −1.6 mm), or the BNST (150 nl; AP + 0.65, ML ± 1.00, DV −4.25). All coordinates are relative to bregma. After injection, the needle was left in place for 10 min before slowly retracting. The incision was sutured, and mice were weighed and monitored to ensure recovery.

### Immunohistochemistry

**Figure 2:**
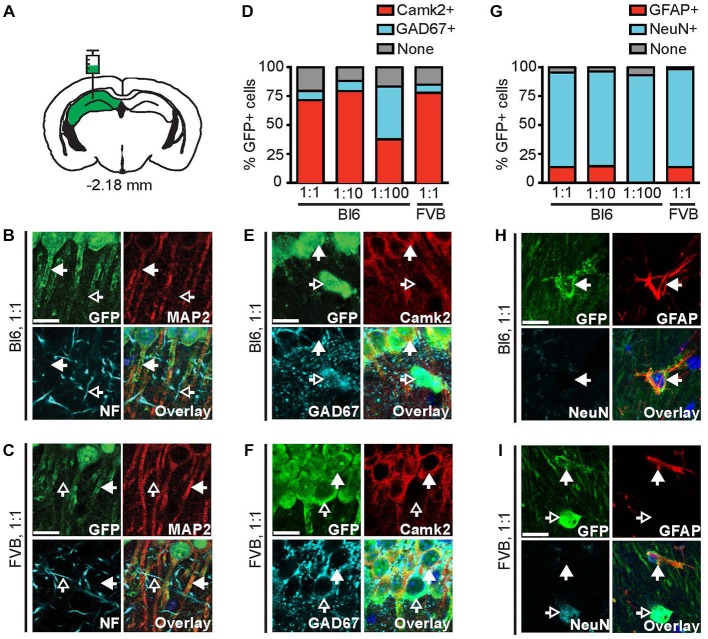
**The 1.3 kb Camk2a promoter does not confer complete cell-type specific expression. (A)** AAV9-Camk2a-TRAP was injected into the hippocampus of wild type C57BL/6J (Bl6) or FVB mice (1:1 viral titer, 2.18 × 10^13^ viral genomes/ml; images of Bl6 1:10 and Bl6 1:100 are shown in Supplementary Figure 1). **(B,C)** Images of the CA1 region of the hippocampus showing immunohistochemical labeling of EGFP-L10a (green), the neuronal somatodendritic marker MAP2 (red), and the axon marker neurofilament (NF, cyan). **(D)** Quantification of the percentage of EGFP-L10a cells in the CA1 region that overlap with Camk2a (red), and GAD67 (cyan) (total number of EGFP-L10a cells analyzed: 1:1 Bl6, *n* = 479, 1:10 Bl6, *n* = 247, 1:100 Bl6, *n* = 27, 1:1 FVB, *n* = 350). **(E,F)** Images of the CA1 region showing immunohistochemical labeling of EGFP-L10a (green), Camk2a (red), and GAD67 (cyan). **(G)** Quantification of the percentage of EGFP-L10a cells in the CA1 region that overlap with the astrocyte marker GFAP (red), and the neuron marker NeuN (cyan) (total number of EGFP-L10a cells analyzed: 1:1 Bl6, *n* = 460, 1:10 Bl6, *n* = 272, 1:100 Bl6, *n* = 29, 1:1 FVB, *n* = 427). **(H,I)** Images of the CA1 region showing immunohistochemical labeling of EGFP-L10a (green), GFAP (red), and NeuN (cyan). Examples of EGFP-L10a expression in NeuN cells for Bl6 1:1 can be found in Supplementary Figure 2J. **(B,C,E,F,H,I)** Scale bars 12.5 μm, filled arrows indicate cells positive for the red channel, open arrows indicate cells positive for the cyan channel, overlay includes DAPI, see Supplementary Figure 2 for the location of images within the CA1 region.

**Figure 3:**
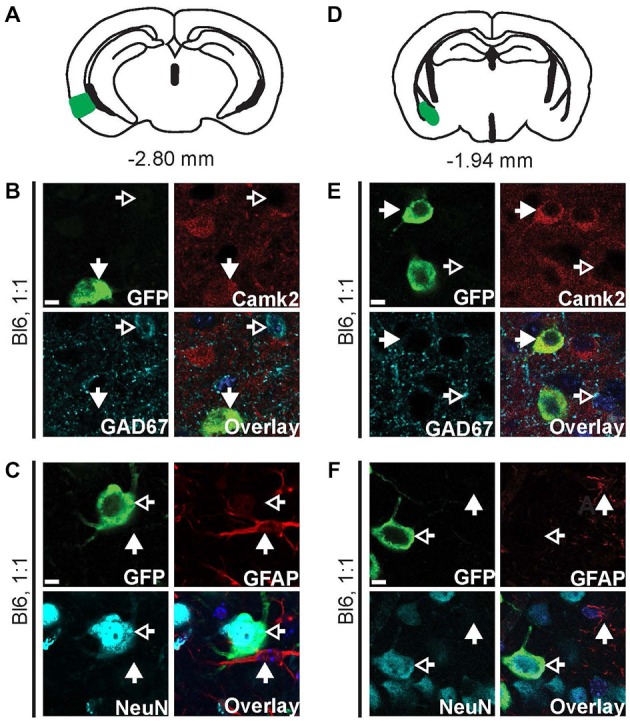
**AAV9-Camk2a-TRAP retrogradely transduces Camk2a-positive neurons that project to the hippocampus.** AAV9-Camk2a-TRAP was injected into the hippocampus of wild type C57BL/6J mice (1:1 viral titer, 2.18 × 10^13^ viral genomes/ml). **(A–C)** Images of the entorhinal cortex (**A**: highlighted in green) showing immunohistochemical labeling of EGFP-L10a (green) with various cell-type markers (**B**: Camk2a, GAD67; **C**: GFAP, NeuN). **(D–F)** Images of the basal amygdala (**D**: highlighted in green) showing immunohistochemical labeling of EGFP-L10a (green) with various cell-type markers (**E**: Camk2a, GAD67; **F**: GFAP, NeuN). **(B,C,E,F)** Scale bars 6.25 μm, filled arrows indicate cells positive for the red channel, open arrows indicate cells positive for the cyan channel, overlay includes DAPI, see Supplementary Figure 3 for the location of images within the entorhinal cortex and basal amygdala.

**Figure 4:**
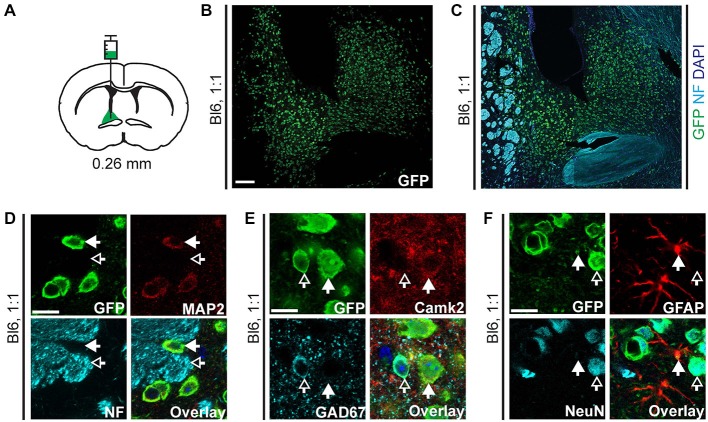
**EGFP-L10a expression in the bed nucleus of the stria terminalis (BNST) after injection of AAV9-Camk2a-TRAP in the BNST. (A)** AAV9-Camk2a-TRAP was injected into an area containing the anterodorsal, anterolateral, and oval subdivisions of the BNST of wild type C57BL/6J mice (1:1 viral titer, 2.18 × 10^13^ viral genomes/ml; bregma: 0.26 mm, targeted BNST subdivisions highlighted in green). **(B,C)** Low magnification images of the BNST showing immunohistochemical labeling of EGFP-L10a (green) by itself **(B)**, and together with the axon marker NF (cyan) and nuclear marker DAPI (blue) **(C)**. White matter tracts demarcating the BNST are easily identified with the NF labeling. Scale bar 100 μm. **(D–F)** High magnification images of the BNST showing immunohistochemical labeling of EGFP-L10a (green) with various subcellular and cell-type markers [**D**: neuronal somatodendritic marker MAP2, axon marker NF; **(E)**: Camk2a, GAD67; **(F)**: GFAP, NeuN]. Scale bars 12.5 μm, filled arrows indicate cells positive for the red channel, open arrows indicate cells positive for the cyan channel, overlay includes DAPI, see Supplementary Figure 4 for the location of images within the BNST.

**Figure 5:**
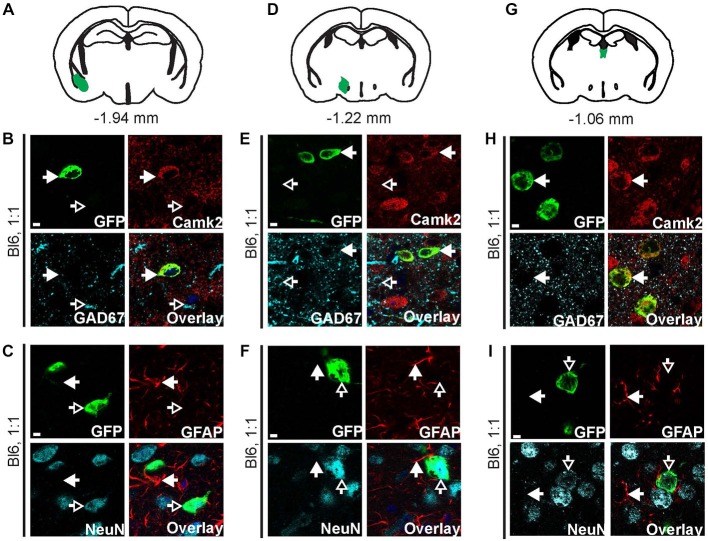
**AAV9-Camk2a-TRAP retrogradely transduces Camk2a-positive neurons that project to the BNST.** AAV9-Camk2a-TRAP was injected into the BNST of wild type C57BL/6J mice (1:1 viral titer, 2.18 × 10^13^ viral genomes/ml). **(A–C)** Images of the basal amygdala (**A**: highlighted in green) showing immunohistochemical labeling of EGFP-L10a (green) with various cell-type markers [**B**: Camk2a, GAD67; **(C)**: GFAP, NeuN]. **(D–F)** Images of the lateral hypothalamus (**D**: highlighted in green) showing immunohistochemical labeling of EGFP-L10a (green) with various cell-type markers [**E**: Camk2a, GAD67; **(F)**: GFAP, NeuN]. **(G–I)** Images of the paraventricular thalamic nucleus (**G**: highlighted in green) showing immunohistochemical labeling of EGFP-L10a (green) with various cell-type markers [**H**: Camk2a, GAD67; **(I)**: GFAP, NeuN]. **(B,C,E,F,H,I)** Scale bars 4 μm, filled arrows indicate cells positive for the red channel, open arrows indicate cells positive for the cyan channel, overlay includes DAPI, see Supplementary Figure 5 for the location of images within the basal amygdala, lateral hypothalamus, and paraventricular thalamic nucleus.

At 14 days after stereotaxic surgery, mice were anesthetized with ketamine/xylazine and transcardially perfused with 4% paraformaldehyde. Brains were extracted, post-fixed in 4% paraformaldehyde for 24 h, transferred to 30% sucrose for 72 h, and flash frozen in isopentane. 20 μm coronal sections were sliced using a cryostat (Leica), and stored in cryoprotectant at −20°C until use. For immunolabeling, free-floating sections were incubated in blocking solution (0.1 M phosphate buffered saline, 5% normal goat serum, and 0.25% Triton X-100) for 1 h at room temperature. Sections were incubated in blocking solution with the following primary antibodies for 72 h at 4°C: chicken anti-GFP (1:500, Aves, GFP-1020), rabbit anti-GFP (1:2000, Invitrogen, A-6455), chicken anti-MAP2 (1:10,000, Abcam, ab5392), mouse anti-neurofilament (1:5000, Abcam, ab24574), rabbit anti-Camk2 (1:2000, Abcam, ab52476), mouse anti-GAD67 (1:10,000, Millipore, MAB5406), chicken anti-GFAP (1:2000, Abcam, ab4674), mouse anti-NeuN (1:2000, Millipore, MAB377). The immunohistochemical staining patterns obtained with these antibodies (Figures 1–5) were in agreement with the known expression patterns of the corresponding proteins, both at the cellular and subcellular level, supporting the specificity of these antibodies. Sections were incubated in the following secondary antibodies for 2 h at room temperature (all from Jackson ImmunoResearch): goat anti-chicken Dylight488 (1:500) or Cy3 (1:1000), goat anti-rabbit Dylight488 (1:1000) or Cy3 (1:2000), goat anti-mouse Dylight647 (1:500). Sections were mounted to glass slides using Prolong Gold Anti-Fade Reagent containing DAPI (Invitrogen), coverslipped, and stored at 4°C until imaging.

### Image Collection and Analysis

**Figure 6:**
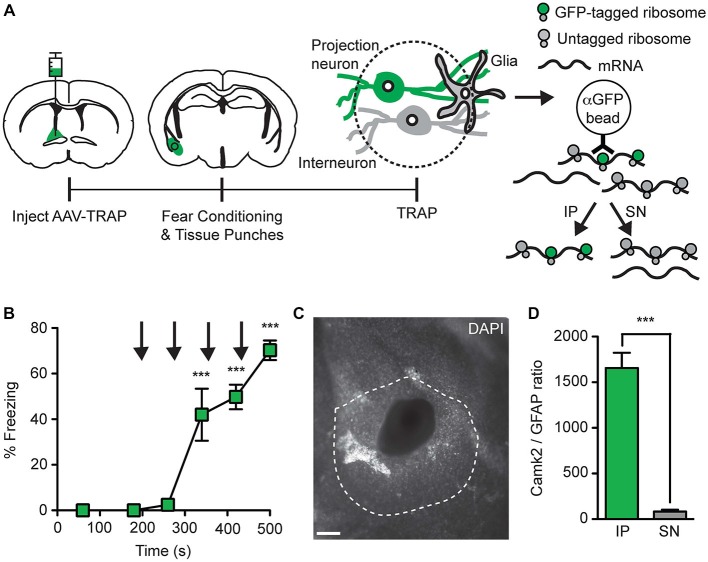
**Collection of ribosome-bound mRNA from basal amygdala neurons that project to the BNST. (A)** AAV9-Camk2a-TRAP was injected into the BNST of wild-type C57BL/6J mice (1:1 viral titer, 2.18 × 10^13^ viral genomes/ml). 14 days later the mice were subjected to a single fear conditioning trial, which was immediately followed by collection of tissue punches from the basal amygdala. Tissue punches were subjected to TRAP, which separates mRNA into immunoprecipitate (IP) and supernatant (SN) fractions using anti-GFP-conjugated magnetic beads. The IP contains mRNA bound to EGFP-L10a containing ribosomes present in retrogradely transduced basal amygdala projection neurons, while the SN contains mRNA from other sources. **(B)** Freezing behavior was determined during fear conditioning (*n* = 3; ***p < 0.001 vs. 60 s by one-way ANOVA plus Newman-Keuls *post hoc* testing; black arrows indicate footshocks). **(C)** Representative image of a basal amygdala tissue punch (white: DAPI; dotted line indicates basal amygdala; scale bar 100 μm). **(D)** Camk2a and GFAP mRNA was quantified in the IP and SN fractions by quantitative polymerase chain reaction (qPCR), followed by calculation of the Camk2a/GFAP ratios in these fractions (*n* = 3; ***p < 0.001 IP vs. SN by paired *t*-test).

A confocal laser-scanning microscope was used for all image acquisition (Nikon A1R), except for Figure 6C where a wide-field epifluorescence microscope was used (Nikon E800). Confocal image stacks were collected with a 2 μm step using 10×, 20×, or 60× objectives. The settings for PMT, laser power, gain, and offset were identical between experimental groups.

### Fear Conditioning

At 14 days after stereotaxic surgery, mice underwent a single contextual fear conditioning trial in a specialized chamber (H10-11RTC, 120W × 100D × 120H; Coulbourn Instruments). The trial lasted 500 s with 2 s, 0.7 mA foot shocks administered at 198, 278, 358 and 438 s. Freezing behavior was measured using Actimetrics FreezeFrame software during two intervals before the first shock (0–60 s, 60–180 s), and during intervals starting 20 s after each of the four shocks (220–260 s, 300–340 s, 380–420 s, and 460–500 s).

### Tissue Dissection for TRAP Analysis

Immediately following fear conditioning, mice were anesthetized with isoflurane and their brains were promptly removed in ice-cold dissection buffer (1 × HBSS, 2.5 mM HEPES-KOH, 35 mM glucose, 4 mM NaHCO3). Brains were frozen in liquid nitrogen, and manually sliced into coronal sections (~1 mm thickness) using a sterile scalpel blade. Brain sections were kept frozen on a cold plate at −10°C (TCP-2D, Thermoelectrics Unlimited), and tissue punches were taken from the basal amygdala using a 24-gauge NIH style neuropunch tool (Fine Science Tools, 18036-24) under a dissection microscope. Coronal sections were placed in 4% paraformaldehyde overnight, moved to 30% sucrose for 24 h, and stained with DAPI. Correct placement of the basal amygdala punch was confirmed for the anterior and posterior faces of each coronal slice. For each mouse, three correctly placed tissue punches (~1 mm thick and ~0.31 mm in diameter) were pooled prior to immunoprecipitation.

### RNA Collection Using TRAP

Collection of RNA was performed using a modified version of the TRAP protocol (Heiman et al., 2008), as described previously (Ainsley et al., 2014; Drane et al., 2014). We have previously verified that this protocol results in low background when brain tissue is used from mice that do not express EGFP-L10a (Ainsley et al., 2014; Drane et al., 2014). Briefly, a monoclonal anti-GFP antibody from the Monoclonal Antibody Core Facility at Memorial Sloan-Kettering Cancer Center (purified form of HtzGFP-19C8) was bound to magnetic epoxy beads (Invitrogen), and the beads were treated with bovine serum albumin to reduce nonspecific binding. For each mouse, three tissue punches were pooled, homogenized, centrifuged to pellet debris, and incubated with the beads at 4°C for 1 h. The supernatant (SN) was saved and the beads were washed five times with a KCl buffer. Trizol LS was used to extract RNA from the beads and the SN followed by back extraction to improve yield. Organic contaminants were removed with butanol and RNA was precipitated with isopropanol, NaOAc, and linear acrylamide overnight at −80°C. After two washes with 80% EtOH, RNA was resuspended in 10 ul of nuclease-free water.

### Reverse Transcription and qPCR

RNA samples were treated with DNase to remove potential contamination with DNA. Expected RNA amounts for each sample were in the picogram range based on a previous study (Ainsley et al., 2014). Because of the low expected amounts of RNA, no RNA was used to perform precise quantification and RIN analysis of the total amount of RNA. Instead equal volume amounts were used for each quantitative polymerase chain reaction (qPCR). Reverse transcription was carried out using Superscript III (Invitrogen) primarily as described by the manufacturer with the exception that both random hexamers and anchored oligo-dT were used for priming. qPCR was performed with SYBR green master mix (Applied Biosystems) on a Mx4000 thermal cycler (Agilent) using the following primer sequences: Camk2a forward primer, 5′-TTTGAGGAACTGGGAAAGGG-3′; Camk2a reverse primer, 5′-CATGGAGTC GGACGATATTGG-3′; GFAP forward primer, 5′-CGGAGACGCATCACCTCTG-3′; GFAP reverse primer, 5′-AGGGAGTGGAGGAGTCATTCG-3′. Camk2a/GFAP ratios for both the immunoprecipitate (IP) and SN fractions were calculated as 2^(^CT^GFAP—^CT^Camk2a)^ (CT: qPCR Cycle Threshold).

## Results

### AAV9 Retrogradely Transduces Entorhinal Cortex Neurons that Project to the Hippocampus

We made a construct in which a 1.3 kb fragment of the Camk2a promoter drives the expression of a fusion protein consisting of EGFP attached to the N-terminus of the ribosomal protein L10a (Figure 1A). As the expression of EGFP-L10a can be used for TRAP (Heiman et al., 2008), we will refer to this construct as Camk2a-TRAP. To express the Camk2a-TRAP construct in the brain, we packaged it in an AAV9. After injection of AAV9-Camk2a-TRAP into the dorsal hippocampus of C56BL/6J mice, we observed widespread expression of EGFP-L10a in the hippocampus (Figures 1B,C). This expression was more sparse when the virus was diluted (Figures 1D,E), and was similar in a different strain of mice (FVB; Figure 1F).

Since both retrograde transport and anterograde trans-synaptic transport of AAV9 has been observed in previous studies (Cearley and Wolfe, 2006, 2007; Masamizu et al., 2011; Löw et al., 2013; Castle et al., 2014a), we analyzed EGFP-L10a expression in the entorhinal cortex, a brain region that has strong reciprocal connections with the hippocampus (van Groen et al., 2003; van Strien et al., 2009). We observed EGFP-L10a expression in the entorhinal cortex that, similar to the hippocampus, was more sparse when the virus was diluted (Figures 1H–K). Entorhinal cortex expression was predominantly observed in layer II, with additional expression in layer III (Figures 1H–K). We observed only sporadic expression in layers V and VI (data not shown). Most entorhinal cortex neurons that project to the hippocampus reside in layers II and III, while most entorhinal cortex neurons that receive input from hippocampal neurons reside in layers V and VI (van Groen et al., 2003; van Strien et al., 2009). Therefore, the observed entorhinal cortex expression pattern indicated that the intra-hippocampal injected AAV9-Camk2a-TRAP was taken up by axon terminals of entorhinal cortex projection neurons, and retrogradely transported to their soma for transcription and translation of EGFP-L10a.

### A 1.3 kb Camk2a Promoter is Insufficient to Confer Complete Cell-Type Specific Expression

To confirm that EGFP-L10a protein expressed by AAV9-Camk2a-TRAP is functional, we determined the subcellular localization of EGFP-L10a. EGFP-L10a protein was present in the soma and dendrites of CA1 pyramidal neurons, but not in axons (Figures 2A–C, Supplementary Figures S1A–C, S2A–E), in agreement with the previously reported somatodendritic localization of ribosomes in neurons (Bodian, 1965; Steward and Levy, 1982; Ostroff et al., 2002). To verify whether the 1.3 kb Camk2a promoter restricted EGFP-L10a expression to neurons that express endogenous Camk2a, we compared EGFP-L10a expression in the hippocampal CA1 region with the expression of endogenous Camk2a protein and several other cell type markers. This revealed that in the CA1 region the majority of EGFP-L10a expressing cells were Camk2a-expressing CA1 pyramidal neurons (Figures 2D–F, Supplementary Figures S1D,E, S2F–I). However, a fraction of EGFP-L10a expression was detected in CA1 cells that do not express Camk2a, including GAD67-positive interneurons (Figures 2D–F, Supplementary Figures S1D,E, S2F–I), and GFAP-positive astrocytes (Figures 2G–I, Supplementary Figures S1F,G, S2J–M). This lack of complete cell-type specificity was observed in both C57BL/6J and FVB mice, and when 10× and 100× diluted virus was injected in C57BL/6J mice (Figures 2D,G). To ensure that the lack of cell-type specificity was not the result of one or more mutations in the 1.3 kb Camk2a promoter, we sequenced the promoter. This confirmed that the 1289 bp Camk2a promoter sequence in AAV9-Camk2a-TRAP completely aligns with a 1289 bp sequence located within the Camk2a promoter region of the mouse genome (chr18: 60, 924, 430–60, 925, 718, UCSC Dec. 2011 mm10 assembly), with the exception of 1 bp at the location of a single-nucleotide polymorphism that is common among mouse strains (dbSNP build 138 rs30301076).

### Retrogradely Transduced Projection Neurons are Camk2a Positive

We next performed cell-type analysis of cells in the entorhinal cortex that expressed EGFP-L10a after injection of AAV9-Camk2a-TRAP in the hippocampus. Within the entorhinal cortex, almost all of the EGFP-L10a expressing cells were Camk2a-positive neurons, with no EGFP-L10a expression found in GAD67-positive interneurons and GFAP-positive astrocytes (Figures 3A–C, Supplementary Figures S3A–C). This pattern is in agreement with a retrograde transduction of neurons that project from layers II-III of the entorhinal cortex to the hippocampus (van Groen et al., 2003; van Strien et al., 2009), as opposed to passive diffusion of the virus from the hippocampus to the entorhinal cortex. In case of passive diffusion, some EGFP-L10a expression would be expected in GAD67-positive interneurons and GFAP-positive astrocytes similar to the observed expression pattern around the injection site in the hippocampus (Figure 2). We also observed EGFP-L10a expressing cells in the basal amygdala, in agreement with the existence of Camk2a-expressing neurons that project from the basal amygdala to the hippocampus (Pitkänen et al., 2000; McDonald et al., 2002). Accordingly, almost all of the EGFP-L10a expressing cells in the basal amygdala were Camk2a-positive neurons, with no EGFP-L10a expression found in GAD67-positive interneurons and GFAP-positive astrocytes (Figures 3D–F, Supplementary Figures S3D–F). These results illustrate the ability of AAV9-Camk2a-TRAP to retrogradely transduce multiple classes of Camk2a-expressing projection neurons.

### AAV9 Retrogradely Transduces Neurons that Project to the Bed Nucleus of the Stria Terminalis

To verify that the retrograde transduction capacity of AAV9-Camk2a-TRAP is not limited to intra-hippocampal injections, we injected the virus in a second brain region. Injection of AAV9-Camk2a-TRAP into the BNST (anterodorsal, anterolateral, and oval subdivisions) resulted in widespread EGFP-L10a expression around the site of injection (Figures 4A–C, Supplementary Figures S4A–D). In agreement with its incorporation into ribosomes, EGFP-L10a expression was somatodendritic without expression in axons (Figure 4D, Supplementary Figure S4B). Similar to the hippocampus, EGFP-L10a expression was not completely cell-type specific. Though many EGFP-L10a expressing cells around the site of injection were Camk2a-positive, EGFP-L10a expression was also observed in GAD67-positive neurons and GFAP-positive astrocytes (Figures 4E,F, Supplementary Figures S4C,D).

We next determined potential retrograde transport of AAV9-Camk2a-TRAP by analyzing EGFP-L10a expression in three brain regions known to contain neurons that project to the BNST: the basal amygdala, the lateral hypothalamus, and the paraventricular nucleus of the thalamus (Arluison et al., 1994; Dong et al., 2001a; Li and Kirouac, 2008). In all three brain regions EGFP-L10a expression was observed (Figure 5, Supplementary Figure S5), and in all three brain regions EGFP-L10a expression was cell-type specific and restricted to Camk2a-expressing neurons (Figure 5, Supplementary Figure S5). Since the BNST subdivisions surrounding the injection site do not contain neurons that project to the basal amygdala (Dong et al., 2001b; Dong and Swanson, 2004, 2006), the observed EGFP-L10a expression in basal amygdala neurons did not result from a previously reported trans-synaptic anterograde transport of AAV9 (Castle et al., 2014a). These data further confirm the retrograde transduction capacity of AAV9-Camk2a-TRAP.

### AAV9-Camk2a-TRAP Enables the Collection of mRNA from Anatomically Defined Projection Neurons

To test whether mRNA can be collected from projection neurons that are retrogradely transduced with AAV9-Camk2a-TRAP, we injected the virus in the BNST and performed TRAP analysis of microdissected basal amygdala tissue (Figure 6A). A critical requirement for the successful application of TRAP is that the mRNA in the cell-type of interest is bound to GFP-tagged ribosomes. Since the level of ribosome-association is expected to increase during increased levels of translation, and since neuronal activation has been reported to increase translation (Kelleher et al., 2004; Costa-Mattioli et al., 2009), we attempted to activate the basal amygdala neurons that project to the BNST immediately before microdissection of the basal amygdala. For this we used contextual fear conditioning, which activates basal amygdala projection neurons (Stanciu et al., 2001; Reijmers et al., 2007). Intra-BNST injection of AAV9-Camk2a-TRAP did not prevent the acquisition of conditioned fear (Figure 6B). The brains were dissected immediately after a single fear conditioning trial, rapidly frozen, and cut into coronal sections. The basal amygdala was microdissected using a tissue puncher, and the correct location of each microdissection was verified with a microscope (Figure 6C). Basal amygdala microdissections were processed for TRAP, and mRNA from both the IP and SN fractions was analyzed with a qPCR of Camk2a and GFAP. We observed a higher Camk2a/GFAP ratio in the IP as compared to the SN, in agreement with the expression of EGFP-L10a in basal amygdala projection neurons that are Camk2a-positive and GFAP-negative (Figures 5A–C, 6D). These data indicate that AAV9-Camk2a-TRAP enables the collection of mRNA from anatomically defined projection neurons.

## Discussion

In this study we performed a detailed histological analysis of mouse brains injected with an AAV9 that expresses EGFP-L10a under control of a 1.3 kb Camk2a promoter (AAV9-Camk2a-TRAP). Two conclusions can be drawn from this analysis. First, the 1.3 kb Camk2a promoter does not confer complete cell-type specific expression around the site of injection, as we observed EGFP-L10a expression in cells that do not express endogenous Camk2a. Second, injection of AAV9-Camk2a-TRAP enables the expression of EGFP-L10a in projection neurons defined by their projection target. This expression can be used for TRAP analysis, as illustrated by the collection of mRNA from retrogradely transduced neurons that project from the basal amygdala to the BNST. Employment of AAV9-Camk2a-TRAP in combination with RNA-Seq should enable the genome-wide molecular profiling of anatomically defined projection neurons in mice, as well as other mammalian species (Masamizu et al., 2011; Löw et al., 2013).

We found that the 1.3 kb Camk2a promoter was insufficient to confer complete cell-type specific expression of EGFP-L10a around the site of virus injection in two different brain regions (hippocampus and BNST). Most EGFP-L10a expression was observed in Camk2a-expressing neurons, in agreement with previous studies that employed the 1.3 kb Camk2a promoter to drive transgene expression in Camk2a-expressing projection neurons in various brain regions (Dittgen et al., 2004; Nathanson et al., 2009; Johansen et al., 2010; Lee et al., 2010; Stuber et al., 2011; Tye et al., 2011; Jennings et al., 2013; Nieh et al., 2015). The EGFP-L10a expression in hippocampal GABAergic interneurons observed in our study is in agreement with previous studies that also reported expression in GABAergic neurons after injection of AAV containing the 1.3 kb Camk2a promoter (Nathanson et al., 2009; Johansen et al., 2010; Jennings et al., 2013; Nieh et al., 2015). Our data clearly establish that the 1.3 kb Camk2a promoter can drive expression in both neuronal and non-neuronal cells that are Camk2a-negative. In contrast, we and others previously found that a transgenic Camk2a promoter of 8.5 kb length is sufficient to confer cell-type specificity in the CA1 region of the hippocampus and in other regions of the brain (Mayford et al., 1996; Ainsley et al., 2014; Drane et al., 2014). This suggests that the 8.5 kb Camk2a promoter contains critical regulatory sequences that are missing in the 1.3 kb Camk2a promoter. Alternatively, complete cell-type specificity might require some form of epigenetic regulation that is missing when the Camk2a promoter is placed in AAV, due to the episomal nature of the AAV genome in transduced cells. It should be noted that the ITR region of AAV has been reported to drive expression in brain cells in the absence of a promoter, though with low efficiency (Haberman et al., 2000). Therefore, it can not be ruled out that the ITR in AAV-Camk2a-TRAP may have contributed to the observed expression of EGFP-L10a in Camk2a-negative cells. Despite being imperfect as a cell-type specific driver, the 1.3 kb Camk2a promoter is useful for achieving high transgene expression in projection neurons that are subsequently analyzed or manipulated outside the sphere of passive virus diffusion. This can be achieved with the retrograde targeting of the soma, as was done in this study, or with the anterograde targeting of axons using a virus that expresses an optogenetic or pharmacogenetic effector protein combined with local administration of the light or drug to the axon terminals only (Lee et al., 2010; Stuber et al., 2011; Tye et al., 2011; Jennings et al., 2013; Stachniak et al., 2014; Nieh et al., 2015).

The ability of AAV9-Camk2a-TRAP to retrogradely transduce projection neurons, and subsequently drive expression of EGFP-L10a in these neurons, opens up the possibility of collecting ribosome-bound mRNA from anatomically defined projection neurons. Accordingly, we were able to collect mRNA from projection neurons defined by the location of their soma in the basal amygdala and the location of their axon terminals in the BNST. Three points need to be considered when employing AAV9-Camk2a-TRAP in future studies. First, though we were able to target a sufficient number of BA to BNST projecting neurons for the successful performance of TRAP, the practical utility of AAV9-Camk2a-TRAP will need to be validated on a case-by-case basis for each new group of projection neurons. Depending on the density of axon terminals, and the efficacy of AAV endocytosis and retrograde transport, different groups of projection neurons are likely to express different amounts of EGFP-L10a, and as a result will be more or less suitable for subsequent TRAP analysis. It should be noted that a high titer AAV9 preparation appears essential for the targeting of sufficient numbers of projection neurons (Figure 1). Even at the highest titer used in our study, the average number of retrogradely transduced BA neurons following injection of AAV9-Camk2a-TRAP into the BNST was less than the average number of retrogradely labeled BA neurons following injection of the retrograde tracer Cholera Toxin B into the BNST (~6.5% for AAV9-Camk2a-TRAP, Supplementary Figure S5C, vs. ~23% for Cholera Toxin B, data not shown). A higher retrograde transduction rate might be achieved by further increasing the viral titer, and by injecting more viral solution (at a lower injection rate to prevent viral spread outside of the target region). Second, the Camk2a promoter, though not conferring complete cell-type specificity, does favor expression in Camk2a expressing neurons (Figure 2). It is therefore unlikely that AAV9-Camk2a-TRAP can be used for sufficient retrograde targeting of projection neurons that do not express Camk2a. This can be resolved by replacing the Camk2a promoter in AAV9-Camk2a-TRAP with a different promoter that is known to drive high expression in the projection neurons of interest. Third, it has become clear that intermingled projection neurons with overlapping projection targets can have different molecular identities (McDonald et al., 2012; Jennings et al., 2013; Nieh et al., 2015). In those cases, the use of our AAV9-Camk2a-TRAP tool might result in the collection of ribosome-bound mRNA from two or more classes of projection neurons that have different molecular profiles. Follow-up experiments that combine a retrograde tracer with multiplex *in situ* hybridization could resolve whether a defined projection pathway is molecularly heterogeneous.

Our AAV9-Camk2a-TRAP tool bears similarity with a related tool that is based on the expression of an EGFP-nanobody tagged ribosomal protein L10a (NBL10: nanobody-L10a) (Ekstrand et al., 2014). By co-expressing NBL10 and EGFP in anatomically defined projection neurons using the retrograde canine adenovirus, ribosomes can be indirectly tagged with EGFP through binding of EGFP to NBL10. This enabled the collection of ribosome-bound mRNA from anatomically defined projection neurons using an immunoprecipitation protocol that is similar to the one used for TRAP (Ekstrand et al., 2014). The NBL10 tool requires the injection of two viruses or the use of a NBL10 transgenic mouse. In contrast, AAV9-Camk2a-TRAP can be readily employed with a single virus injection in any mouse model without additional breeding. This will be especially useful for the molecular profiling of projection neurons in genetic mouse models for brain disorders. On the other hand, the dual-component nature of the NBL10/EGFP system enables an intersectional strategy for achieving a higher level of cell-type specific targeting. Future studies comparing the two tools side-by-side will be useful for determining conditions where the use of one of these tools is preferable. An important consideration should be that the two tools are based on two different retrograde viruses, AAV9 vs. canine adenovirus. Though these two viruses seem to use similar machinery for retrograde axonal transport in cultured neurons (Salinas et al., 2009; Castle et al., 2014b), it is possible that they have different retrograde transduction properties *in vivo*. A future study that directly compares the *in vivo* retrograde transduction efficacies of AAV9 with canine adenovirus, and other retrograde viruses such as lentivirus pseudotyped with rabies virus glycoprotein (Mazarakis et al., 2001), would therefore be extremely useful.

There are two general points of consideration when molecular profiling is done through the employment of epitope-tagged ribosomal proteins such as EGFP-L10a, NBL10, or other variants (Heiman et al., 2008; Sanz et al., 2009; Ekstrand et al., 2014). First, use of an antibody is needed to IP the epitope-tagged ribosomes and associated mRNA. Since antibodies are rarely completely specific, some mRNA-protein complexes might be collected without being associated with an epitope-tagged ribosome. The resulting non-specific background mRNA signal in the IP fraction can be filtered out *post hoc* by focusing on mRNAs that are enriched in the IP fraction vs. the SN or input fraction (Heiman et al., 2008; Sanz et al., 2009; Dougherty et al., 2010; Ainsley et al., 2014; Drane et al., 2014; Ekstrand et al., 2014). Second, epitope-tagged ribosomes enable the collection of translated, but not untranslated, mRNA. As the translational state of mRNAs can be regulated by neuronal activity (Kelleher et al., 2004; Costa-Mattioli et al., 2009), it is important to control the various environmental and internal factors that could alter the activation state of the projection neurons that express the epitope-tagged ribosomes. This is illustrated by previous TRAP studies that observed changes in the pool of ribosome-bound mRNA when the activation state of the neurons was altered (Heiman et al., 2008; Schmidt et al., 2012; Huang et al., 2013; Ainsley et al., 2014). In our current study we controlled the activation state of BA to BNST projecting neurons by performing TRAP analysis on mice that were sacrificed at the same time-point after a single fear conditioning trial. It will be of interest to perform future studies that employ AAV9-Camk2a-TRAP in separate groups of mice with different activation states, including mice at baseline. Comparisons between these groups could identify proteins whose synthesis is activity-regulated within an anatomically defined class of projection neurons, which could lead to the discovery of novel molecular mechanisms that support information storage in these neurons.

Obtaining a complete molecular profile of a defined class of projection neurons will require the employment of various methods, including methods that are not based on epitope-tagged ribosomes. For example, dissociation of neurons from brain tissue enables the collection and analysis of all types of RNA, including untranslated mRNA and non-coding RNA. Dissociating neurons that are labeled with a retrograde tracer (Cholera Toxin B, Fluoro-Gold, latex microspheres) enables the collection of all types of RNA from target-defined projection neurons (Arlotta et al., 2005; Sugino et al., 2006; Dugas et al., 2008). However, dissociating neurons from brain tissue causes the loss of the RNA pool that is located in neuronal processes such as dendrites, which can contain more than half of the cellular volume of a neuron (Ulfhake and Kellerth, 1981; Holt and Schuman, 2013). Two recent studies reported the RNA-Seq analysis of transcriptomes collected from single neurons from mouse and human brain (Darmanis et al., 2015; Zeisel et al., 2015). These single-cell methods enable the molecular profiling of projection neurons with high specificity, but still require dissociation of neurons. An alternative to sequencing RNA collected through epitope-tagging or the dissociation of neurons is provided by *in situ* detection of RNA. Highly multiplexed *in situ* detection of RNA in neurons has recently become possible, thereby enabling molecular profiling of neurons while retaining the precise subcellular localization of the RNAs within the soma and processes (Lee et al., 2014; Chen et al., 2015). It remains to be determined whether these multiplex *in situ* methods can achieve the same sensitivity as RNA-Seq for the quantitative detection of low abundance mRNA.

In conclusion, we report an AAV9-Camk2a-TRAP tool that can retrogradely transduce projection neurons in the mouse brain after its injection at the site of axon terminals, resulting in EGFP-tagged ribosomes in projection neurons defined by their projection target. This enabled the collection of ribosome-bound mRNA from basal amygdala neurons that project to the BNST. As AAV9 has been reported to retrogradely transduce neurons in rat and monkey brains (Masamizu et al., 2011; Löw et al., 2013), we anticipate that the AAV9-Camk2a-TRAP tool can be used for the molecular profiling of anatomically defined projection neurons in a wide variety of mammalian species. In addition, future studies can employ AAV9-Camk2a-TRAP to perform genome-wide analyses of ribosome-bound mRNA collected from anatomically defined projection neurons after various experimental manipulations, which might shed light on protein synthesis events that support information storage in projection neurons.

## Conflict of Interest Statement

The authors declare that the research was conducted in the absence of any commercial or financial relationships that could be construed as a potential conflict of interest.

## References

[B1] Ainsley J. A. Drane L. Jacobs J. Kittelberger K. A. Reijmers L. G. (2014). Functionally diverse dendritic mRNAs rapidly associate with ribosomes following a novel experience. Nat. Commun. 5:4510. 10.1038/ncomms5510 25072471 PMC4160876

[B2] Arlotta P. Molyneaux B. J. Chen J. Inoue J. Kominami R. Macklis J. D. (2005). Neuronal subtype-specific genes that control corticospinal motor neuron development in vivo. Neuron 45, 207–221. 10.1016/j.neuron.2004.12.036 15664173

[B3] Arluison M. Brochier G. Vankova M. Leviel V. Villalobos J. Tramu G. (1994). Demonstration of peptidergic afferents to the bed nucleus of the stria terminalis using local injections of colchicine. A combined immunohistochemical and retrograde tracing study. Brain Res. Bull. 34, 319–337. 10.1016/0361-9230(94)90026-4 7521777

[B4] Bodian D. (1965). A Suggestive Relationship of Nerve Cell Rna with Specific Synaptic Sites. Proc. Natl. Acad. Sci. U S A 53, 418–425. 10.1073/pnas.53.2.418 14294076 PMC219529

[B5] Castle M. J. Gershenson Z. T. Giles A. R. Holzbaur E. L. Wolfe J. H. (2014a). Adeno-associated virus serotypes 1, 8 and 9 share conserved mechanisms for anterograde and retrograde axonal transport. Hum. Gene. Ther. 25, 705–720. 10.1089/hum.2013.189 24694006 PMC4137353

[B6] Castle M. J. Perlson E. Holzbaur E. L. F. Wolfe J. H. (2014b). Long-distance axonal transport of AAV9 is driven by dynein and kinesin-2 and is trafficked in a highly motile rab7-positive compartment. Mol. Ther. 22, 554–566. 10.1038/mt.2013.237 24100640 PMC3944332

[B7] Cearley C. N. Wolfe J. H. (2006). Transduction characteristics of adeno-associated virus vectors expressing cap Serotypes 7, 8, 9 and rh10 in the mouse brain. Mol. Ther. 13, 528–537. 10.1016/j.ymthe.2005.11.015 16413228

[B8] Cearley C. N. Wolfe J. H. (2007). A single injection of an adeno-associated virus vector into nuclei with divergent connections results in widespread vector distribution in the brain and global correction of a neurogenetic disease. J. Neurosci. 27, 9928–9940. 10.1523/jneurosci.2185-07.2007 17855607 PMC6672652

[B9] Chen K. H. Boettiger A. N. Moffitt J. R. Wang S. Zhuang X. (2015). Spatially resolved, highly multiplexed RNA profiling in single cells. Science 348:aaa6090. 10.1126/science.aaa6090 25858977 PMC4662681

[B10] Costa-Mattioli M. Sossin W. S. Klann E. Sonenberg N. (2009). Translational Control of long-lasting synaptic plasticity and memory. Neuron 61, 10–26. 10.1016/j.neuron.2008.10.055 19146809 PMC5154738

[B11] Darmanis S. Sloan S. A. Zhang Y. Enge M. Caneda C. Shuer L. M.. (2015). A survey of human brain transcriptome diversity at the single cell level. Proc. Natl. Acad. Sci. U S A 112, 7285–7290. 10.1073/pnas.1507125112 26060301 PMC4466750

[B12] Dittgen T. Nimmerjahn A. Komai S. Licznerski P. Waters J. Margrie T. W.. (2004). Lentivirus-based genetic manipulations of cortical neurons and their optical and electrophysiological monitoring *in vivo*. Proc. Natl. Acad. Sci. U S A 101, 18206–18211. 10.1073/pnas.0407976101 15608064 PMC539748

[B13] Dong H. W. Petrovich G. D. Swanson L. W. (2001a). Topography of projections from amygdala to bed nuclei of the stria terminalis. Brain Res. Brain Res. Rev. 38, 192–246. 10.1016/s0165-0173(01)00079-0 11750933

[B14] Dong H. W. Petrovich G. D. Watts A. G. Swanson L. W. (2001b). Basic organization of projections from the oval and fusiform nuclei of the bed nuclei of the stria terminalis in adult rat brain. J. Comp. Neurol. 436, 430–455. 10.1002/cne.1079 11447588

[B15] Dong H. W. Swanson L. W. (2004). Organization of axonal projections from the anterolateral area of the bed nuclei of the stria terminalis. J. Comp. Neurol. 468, 277–298. 10.1002/cne.10949 14648685

[B16] Dong H. W. Swanson L. W. (2006). Projections from bed nuclei of the stria terminalis, anteromedial area: cerebral hemisphere integration of neuroendocrine, autonomic and behavioral aspects of energy balance. J. Comp. Neurol. 494, 142–178. 10.1002/cne.20788 16304685 PMC2563961

[B17] Dougherty J. D. Schmidt E. F. Nakajima M. Heintz N. (2010). Analytical approaches to RNA profiling data for the identification of genes enriched in specific cells. Nucleic Acids Res. 38, 4218–4230. 10.1093/nar/gkq130 20308160 PMC2910036

[B18] Doyle J. P. Dougherty J. D. Heiman M. Schmidt E. F. Stevens T. R. Ma G.. (2008). Application of a translational profiling approach for the comparative analysis of CNS cell types. Cell 135, 749–762. 10.3410/f.1127155.584306 19013282 PMC2763427

[B19] Drane L. Ainsley J. A. Mayford M. R. Reijmers L. G. (2014). A transgenic mouse line for collecting ribosome-bound mRNA using the tetracycline transactivator system. Front. Mol. Neurosci. 7:82. 10.3389/fnmol.2014.00082 25400545 PMC4212621

[B20] Dugas J. C. Mandemakers W. Rogers M. Ibrahim A. Daneman R. Barres B. A. (2008). A novel purification method for CNS projection neurons leads to the identification of brain vascular cells as a source of trophic support for corticospinal motor neurons. J. Neurosci. 28, 8294–8305. 10.1523/jneurosci.2010-08.2008 18701692 PMC2567869

[B21] Ekstrand M. I. Nectow A. R. Knight Z. A. Latcha K. N. Pomeranz L. E. Friedman J. M. (2014). Molecular profiling of neurons based on connectivity. Cell 157, 1230–1242. 10.1016/j.cell.2014.03.059 24855954 PMC4854627

[B22] Haberman R. P. McCown T. J. Samulski R. J. (2000). Novel transcriptional regulatory signals in the adeno-associated virus terminal repeat A/D junction element. J. Virol. 74, 8732–8739. 10.1128/jvi.74.18.8732-8739.2000 10954575 PMC116385

[B23] Heiman M. Schaefer A. Gong S. Peterson J. D. Day M. Ramsey K. E.. (2008). A translational profiling approach for the molecular characterization of CNS cell types. Cell 135, 738–748. 10.1016/j.cell.2008.10.028 19013281 PMC2696821

[B24] Holt C. E. Schuman E. M. (2013). The central dogma decentralized: new perspectives on RNA function and local translation in neurons. Neuron 80, 648–657. 10.1016/j.neuron.2013.10.036 24183017 PMC3820025

[B25] Huang Y. Ainsley J. A. Reijmers L. G. Jackson F. R. (2013). Translational profiling of clock cells reveals circadianly synchronized protein synthesis. PLoS. Biol. 11:e1001703. 10.1371/journal.pbio.1001703 24348200 PMC3864454

[B26] Jennings J. H. Sparta D. R. Stamatakis A. M. Ung R. L. Pleil K. E. Kash T. L.. (2013). Distinct extended amygdala circuits for divergent motivational states. Nature 496, 224–228. 10.1038/nature12041 23515155 PMC3778934

[B27] Johansen J. P. Hamanaka H. Monfils M. H. Behnia R. Deisseroth K. Blair H. T.. (2010). Optical activation of lateral amygdala pyramidal cells instructs associative fear learning. Proc. Natl. Acad. Sci. U S A 107, 12692–12697. 10.1073/pnas.1002418107 20615999 PMC2906568

[B28] Kamme F. Salunga R. Yu J. Tran D. T. Zhu J. Luo L.. (2003). Single-cell microarray analysis in hippocampus CA1: demonstration and validation of cellular heterogeneity. J. Neurosci. 23, 3607–3615. 12736331 10.1523/JNEUROSCI.23-09-03607.2003 PMC6742179

[B29] Kelleher R. J. Govindarajan A. Jung H. Y. Kang H. Tonegawa S. (2004). Translational control by MAPK signaling in long-term synaptic plasticity and memory. Cell 116, 467–479. 10.1016/s0092-8674(04)00115-1 15016380

[B30] Lambolez B. Audinat E. Bochet P. Crépel F. Rossier J. (1992). AMPA receptor subunits expressed by single purkinje cells. Neuron 9, 247–258. 10.1016/0896-6273(92)90164-9 1323310

[B31] Lee J. H. Daugharthy E. R. Scheiman J. Kalhor R. Yang J. L. Ferrante T. C.. (2014). Highly multiplexed subcellular RNA sequencing *in situ*. Science 343, 1360–1363. 10.1126/science.1250212 24578530 PMC4140943

[B32] Lee J. H. Durand R. Gradinaru V. Zhang F. Goshen I. Kim D. S.. (2010). Global and local fMRI signals driven by neurons defined optogenetically by type and wiring. Nature 465, 788–792. 10.1038/nature09108 20473285 PMC3177305

[B33] Li S. Kirouac G. J. (2008). Projections from the paraventricular nucleus of the thalamus to the forebrain, with special emphasis on the extended amygdala. J. Comp. Neurol. 506, 263–287. 10.1002/cne.21741 18022956

[B34] Löw K. Aebischer P. Schneider B. L. (2013). Direct and retrograde transduction of nigral neurons with AAV6, 8 and 9 and intraneuronal persistence of viral particles. Hum. Gene. Ther. 24, 613–629. 10.1089/hum.2012.174 23600720 PMC3689167

[B35] Masamizu Y. Okada T. Kawasaki K. Ishibashi H. Yuasa S. Takeda S.. (2011). Local and retrograde gene transfer into primate neuronal pathways via adeno-associated virus serotype 8 and 9. Neuroscience 193, 249–258. 10.1016/j.neuroscience.2011.06.080 21782903

[B36] Mayford M. Bach M. E. Huang Y. Y. Wang L. Hawkins R. D. Kandel E. R. (1996). Control of memory formation through regulated expression of a CaMKII transgene. Science 274, 1678–1683. 10.1126/science.274.5293.1678 8939850

[B37] Mazarakis N. D. Azzouz M. Rohll J. B. Ellard F. M. Wilkes F. J. Olsen A. L.. (2001). Rabies virus glycoprotein pseudotyping of lentiviral vectors enables retrograde axonal transport and access to the nervous system after peripheral delivery. Hum. Mol. Genet. 10, 2109–2121. 10.1093/hmg/10.19.2109 11590128

[B38] McDonald A. J. Mascagni F. Zaric V. (2012). Subpopulations of somatostatin-immunoreactive nonpyramidal neurons in the amygdala and adjacent external capsule project to the basal forebrain: evidence for the existence of GABAergic projection neurons in the cortical nuclei and basolateral nuclear complex. Front. Neural Circuits 6:46. 10.3389/fncir.2012.00046 22837739 PMC3402756

[B39] McDonald A. J. Muller J. F. Mascagni F. (2002). GABAergic innervation of alpha type II calcium/calmodulin-dependent protein kinase immunoreactive pyramidal neurons in the rat basolateral amygdala. J. Comp. Neurol. 446, 199–218. 10.1002/cne.10204 11932937

[B40] Nathanson J. L. Yanagawa Y. Obata K. Callaway E. M. (2009). Preferential labeling of inhibitory and excitatory cortical neurons by endogenous tropism of adeno-associated virus and lentivirus vectors. Neuroscience 161, 441–450. 10.1016/j.neuroscience.2009.03.032 19318117 PMC2728494

[B41] Nieh E. H. Matthews G. A. Allsop S. A. Presbrey K. N. Leppla C. A. Wichmann R.. (2015). Decoding Neural Circuits that Control Compulsive Sucrose Seeking. Cell 160, 528–541. 10.1016/j.cell.2015.01.003 25635460 PMC4312417

[B42] Okaty B. W. Sugino K. Nelson S. B. (2011). Cell type-specific transcriptomics in the brain. J. Neurosci. 31, 6939–6943. 10.1523/jneurosci.0626-11.2011 21562254 PMC3142746

[B43] Ostroff L. E. Fiala J. C. Allwardt B. Harris K. M. (2002). Polyribosomes redistribute from dendritic shafts into spines with enlarged synapses during LTP in developing rat hippocampal slices. Neuron 35, 535–545. 10.1016/S0896-6273(02)00785-7 12165474

[B44] Paxinos G. Franklin K. B. J. (2001). The Mouse Brain in Stereotaxic Coordinates. 2nd Edn. San Diego, CA: Academic Press.

[B45] Pitkänen A. Pikkarainen M. Nurminen N. Ylinen A. (2000). Reciprocal connections between the amygdala and the hippocampal formation, perirhinal cortex and postrhinal cortex in rat:A review. Ann. N Y Acad. Sci. 911, 369–391. 10.1111/j.1749-6632.2000.tb06738.x 10911886

[B46] Reijmers L. G. Perkins B. L. Matsuo N. Mayford M. (2007). Localization of a stable neural correlate of associative memory. Science 317, 1230–1233. 10.1126/science.1143839 17761885

[B47] Salinas S. Bilsland L. G. Henaff D. Weston A. E. Keriel A. Schiavo G.. (2009). CAR-Associated vesicular transport of an adenovirus in motor neuron axons. PLoS Pathog. 5:e1000442. 10.1371/journal.ppat.1000442 19461877 PMC2677547

[B48] Sanz E. Yang L. Su T. Morris D. R. McKnight G. S. Amieux P. S. (2009). Cell-type-specific isolation of ribosome-associated mRNA from complex tissues. Proc. Natl. Acad. Sci. U S A 106, 13939–13944. 10.1073/pnas.0907143106 19666516 PMC2728999

[B49] Schmidt E. F. Warner-Schmidt J. L. Otopalik B. G. Pickett S. B. Greengard P. Heintz N. (2012). Identification of the cortical neurons that mediate antidepressant responses. Cell 149, 1152–1163. 10.1016/j.cell.2012.03.038 22632977 PMC3397430

[B50] Shepherd G. M. G. (2013). Corticostriatal connectivity and its role in disease. Nat. Rev. Neurosci. 14, 278–291. 10.1038/nrn3469 23511908 PMC4096337

[B51] Stachniak T. J. Ghosh A. Sternson S. M. (2014). Chemogenetic synaptic silencing of neural circuits localizes a hypothalamus→midbrain pathway for feeding behavior. Neuron 82, 797–808. 10.1016/j.neuron.2014.04.008 24768300 PMC4306349

[B52] Stanciu M. Radulovic J. Spiess J. (2001). Phosphorylated cAMP response element binding protein in the mouse brain after fear conditioning: relationship to Fos production. Brain Res. Mol. Brain Res. 94, 15–24. 10.1016/s0169-328x(01)00174-7 11597761

[B53] Steward O. Levy W. B. (1982). Preferential localization of polyribosomes under the base of dendritic spines in granule cells of the dentate gyrus. J. Neurosci. 2, 284–291. 7062109 10.1523/JNEUROSCI.02-03-00284.1982 PMC6564334

[B54] Stuber G. D. Sparta D. R. Stamatakis A. M. van Leeuwen W. A. Hardjoprajitno J. E. Cho S.. (2011). Excitatory transmission from the amygdala to nucleus accumbens facilitates reward seeking. Nature 475, 377–380. 10.1038/nature10194 21716290 PMC3775282

[B55] Sugino K. Hempel C. M. Miller M. N. Hattox A. M. Shapiro P. Wu C.. (2006). Molecular taxonomy of major neuronal classes in the adult mouse forebrain. Nat. Neurosci. 9, 99–107. 10.1038/nn1618 16369481

[B56] Tye K. M. Prakash R. Kim S. Y. Fenno L. E. Grosenick L. Zarabi H.. (2011). Amygdala circuitry mediating reversible and bidirectional control of anxiety. Nature 471, 358–362. 10.1038/nature09820 21389985 PMC3154022

[B57] Ulfhake B. Kellerth J. O. (1981). A quantitative light microscopic study of the dendrites of cat spinal alpha-motoneurons after intracellular staining with horseradish peroxidase. J. Comp. Neurol. 202, 571–583. 10.1002/cne.902020410 7298916

[B58] Van Gelder R. N. von Zastrow M. E. Yool A. Dement W. C. Barchas J. D. Eberwine J. H. (1990). Amplified RNA synthesized from limited quantities of heterogeneous cDNA. Proc. Natl. Acad. Sci. U S A 87, 1663–1667. 10.1073/pnas.87.5.1663 1689846 PMC53542

[B59] van Groen T. Miettinen P. Kadish I. (2003). The entorhinal cortex of the mouse: organization of the projection to the hippocampal formation. Hippocampus 13, 133–149. 10.1002/hipo.10037 12625464

[B60] van Strien N. M. Cappaert N. L. M. Witter M. P. (2009). The anatomy of memory: an interactive overview of the parahippocampal-hippocampal network. Nat. Rev. Neurosci. 10, 272–282. 10.1038/nrn2614 19300446

[B61] Zeisel A. Muñoz-Manchado A. B. Codeluppi S. Lönnerberg P. La Manno G. Juréus A.. (2015). Cell types in the mouse cortex and hippocampus revealed by single-cell RNA-seq. Science 347, 1138–1142. 10.1126/science.aaa1934 25700174

