# Supplementary material for: A retrograde adeno-associated virus for collecting ribosome-bound mRNA from anatomically defined projection neurons
Source: Front Mol Neurosci. 2015 Sep 24;8:56. doi: 10.3389/fnmol.2015.00056 (PMC4617378; doi:10.3389/fnmol.2015.00056)
Supplement: Supplementary file 1 [file Presentation_1.pdf]

Supplementary Figures 1-5

A retrograde adeno-associated virus for collecting ribosome-bound mRNA from anatomically defined projection neurons

Denise R. Cook-Snyder[1,2†], Alexander Jones[1,3†], and Leon G. Reijmers[1*]

[1]Department of Neuroscience, School of Medicine, Tufts University, Boston, MA, USA
[2]Department of Biology and Neuroscience Program, Carthage College, Kenosha, WI, USA
[3]Graduate Program in Neuroscience, Sackler School of Graduate Biomedical Sciences, Tufts University, Boston, MA, USA

***Correspondence**: Leon G. Reijmers, Department of Neuroscience, School of Medicine, Tufts University, 136 Harrison Avenue, Boston, MA 02111, USA
e-mail: leon.reijmers@tufts.edu

†These authors have equally contributed to this work.

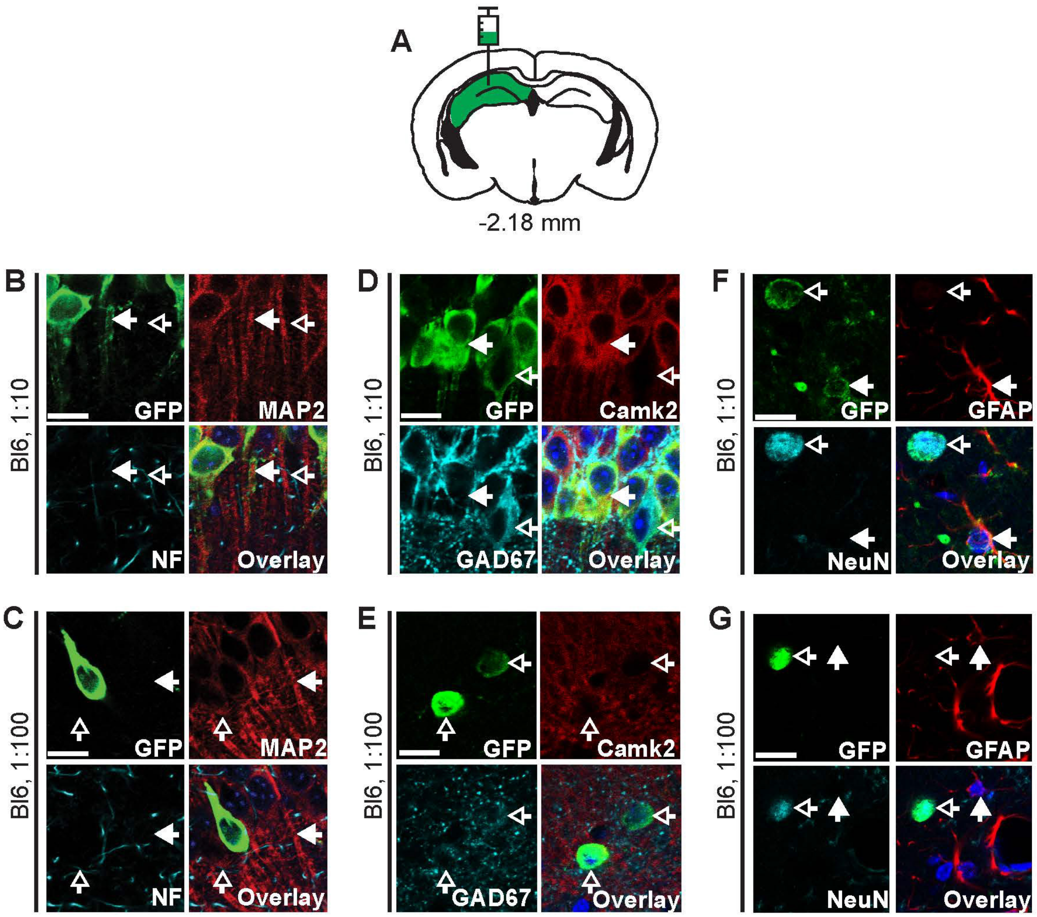

Supplementary Figure 1. EGFP-L10a expression in the CA1 region after injection of diluted AAV9-Camk2a-TRAP in the hippocampus. (A) AAV9-Camk2a-TRAP was injected into the hippocampus of wild type C57BL/6J mice (Bl6 1:10, 2.18 x 1012 viral genomes / ml; Bl6 1:100, 2.18 x 1011 viral genomes / ml; images of 1:1 are shown in Figure 2). **(B-G)** Images of the CA1 region of the hippocampus showing immunohistochemical labeling of EGFP-L10a (green) with various subcellular and cell-type markers (**B-C**: neuronal somatodendritic marker MAP2, axon marker NF; **D-E:** Camk2a, GAD67; **F-G:** GFAP, NeuN). Example of EGFP-L10a expression in a Camk2a-expressing cell for Bl6 1:100 can be found in Supplementary Figure 2I. No EGFP-L10a expression was observed in GFAP-expressing cells for Bl6 1:100 (also see Figure 2G). Scale bars 12.5 μm, filled arrows indicate cells positive for the red channel, open arrows indicate cells positive for the cyan channel, overlay includes DAPI, see Supplementary Figure 2 for the location of images within the CA1 region.

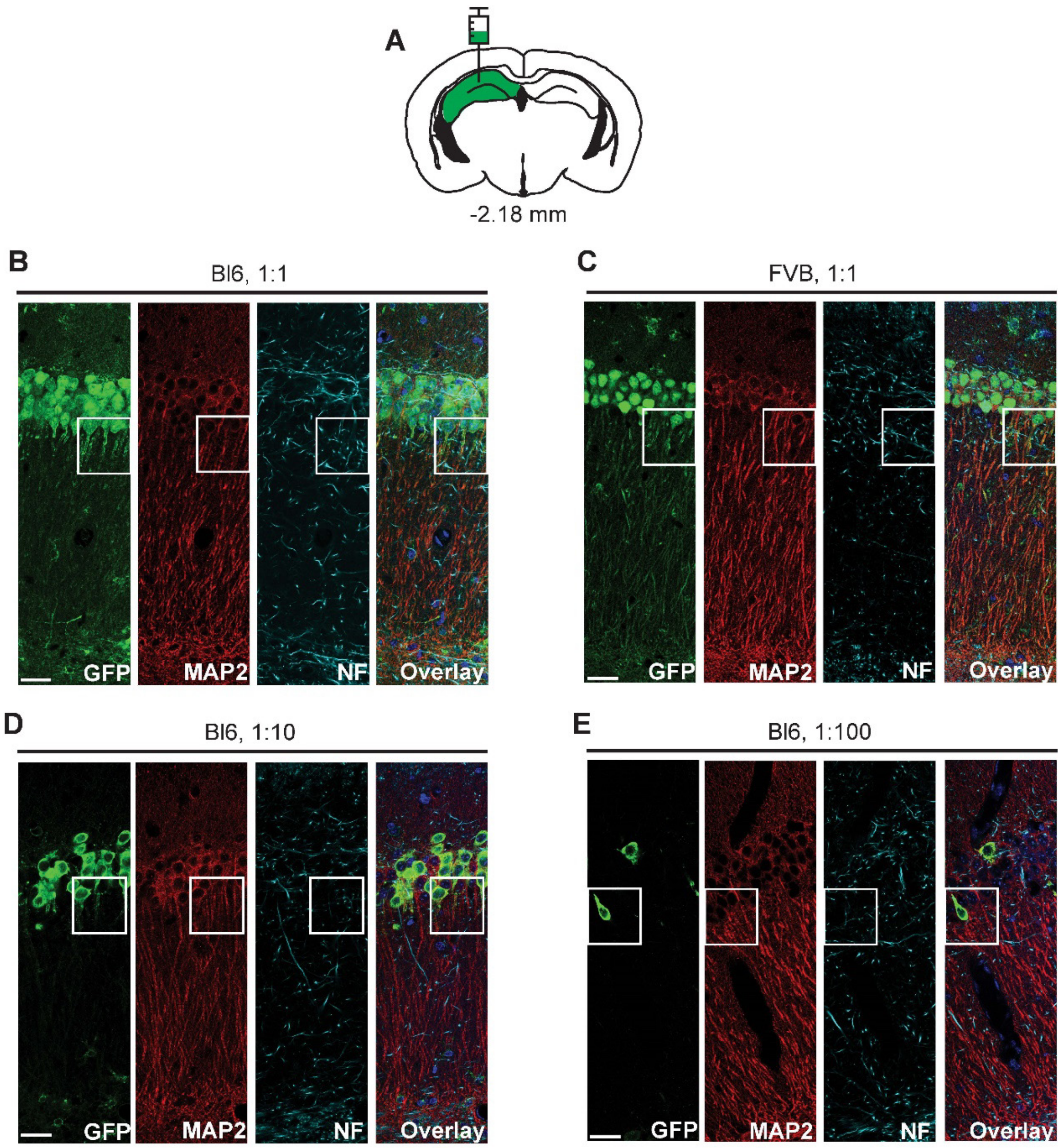

Supplementary Figure 2. Low magnification images of the CA1 region after injection of AAV9-Camk2a-TRAP in the hippocampus. (A) AAV9-Camk2a-TRAP was injected into the hippocampus (highlighted in green) of wild type C57BL/6J or FVB mice using three different dilutions of the virus (1:1, 2.18 x 10^{13} viral genomes / ml; 1:10, 2.18 x 10^{12} viral genomes / ml; 1:100, 2.18 x 10^{11} viral genomes / ml). **(B-M)** Low magnification images of the CA1 region showing immunohistochemical labeling of EGFP-L10a (green) with various subcellular and cell-type markers (**B-E**: neuronal somatodendritic marker MAP2, axon marker NF; **F-I:** Camk2a, GAD67; **J-M:** GFAP, NeuN). Scale bars 25 µm, overlay includes DAPI, boxed areas indicate the location of the high magnification images shown in Figure 2 and Supplementary Figure 1.

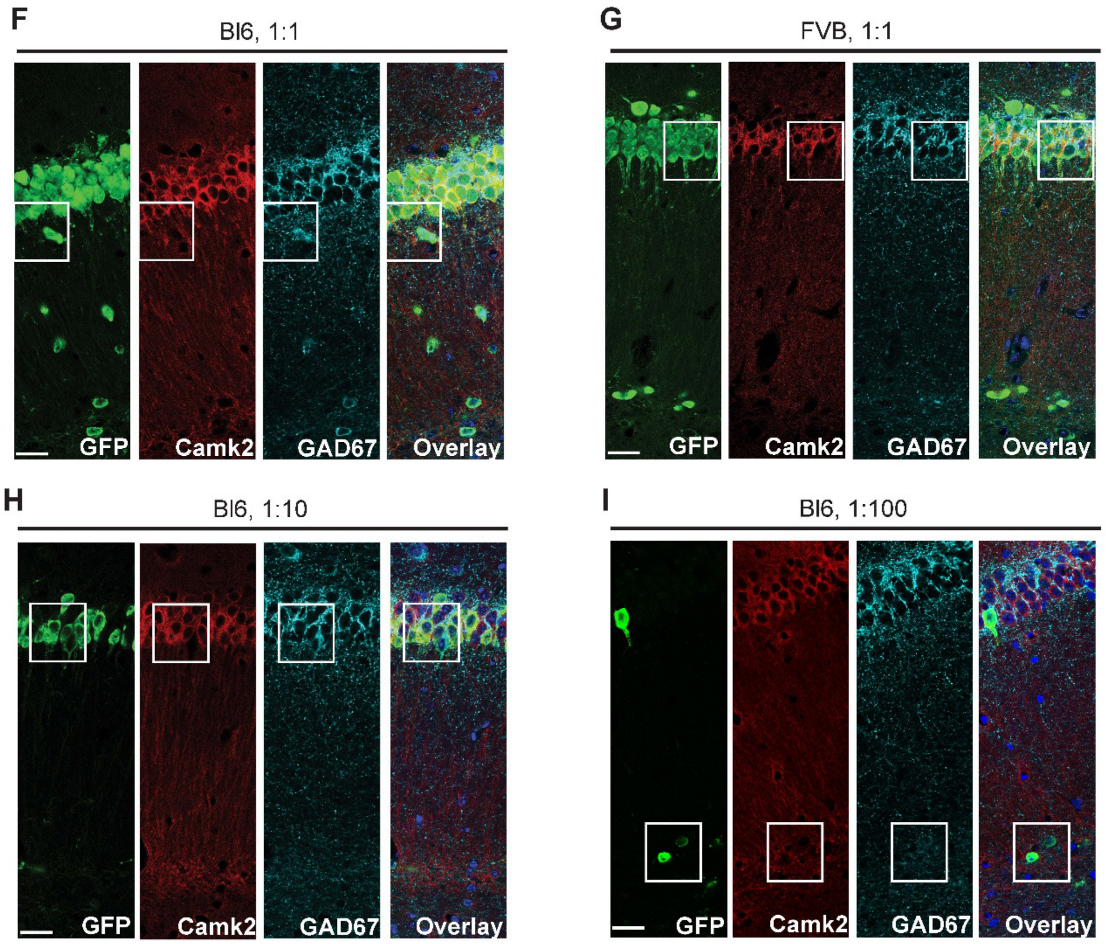

Supplementary Figure 2 (continued).

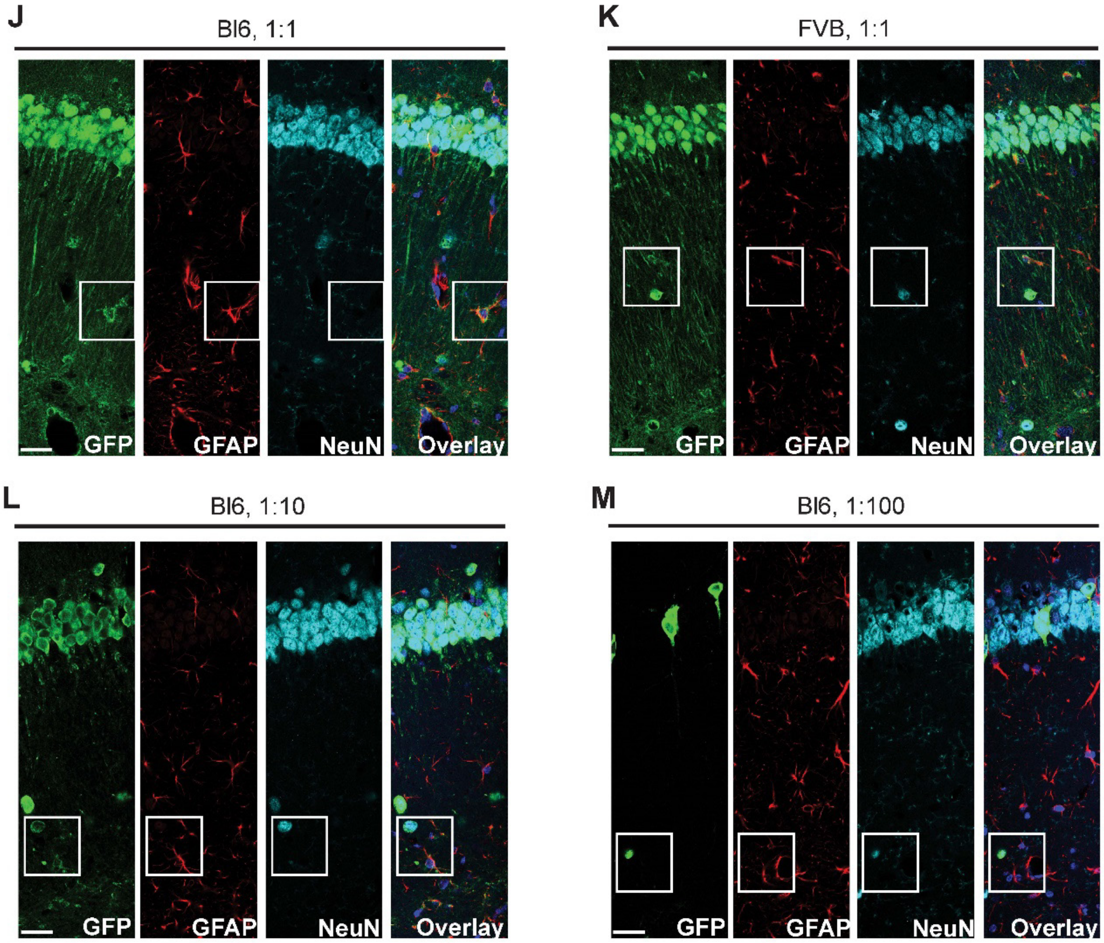

Supplementary Figure 2 (continued).

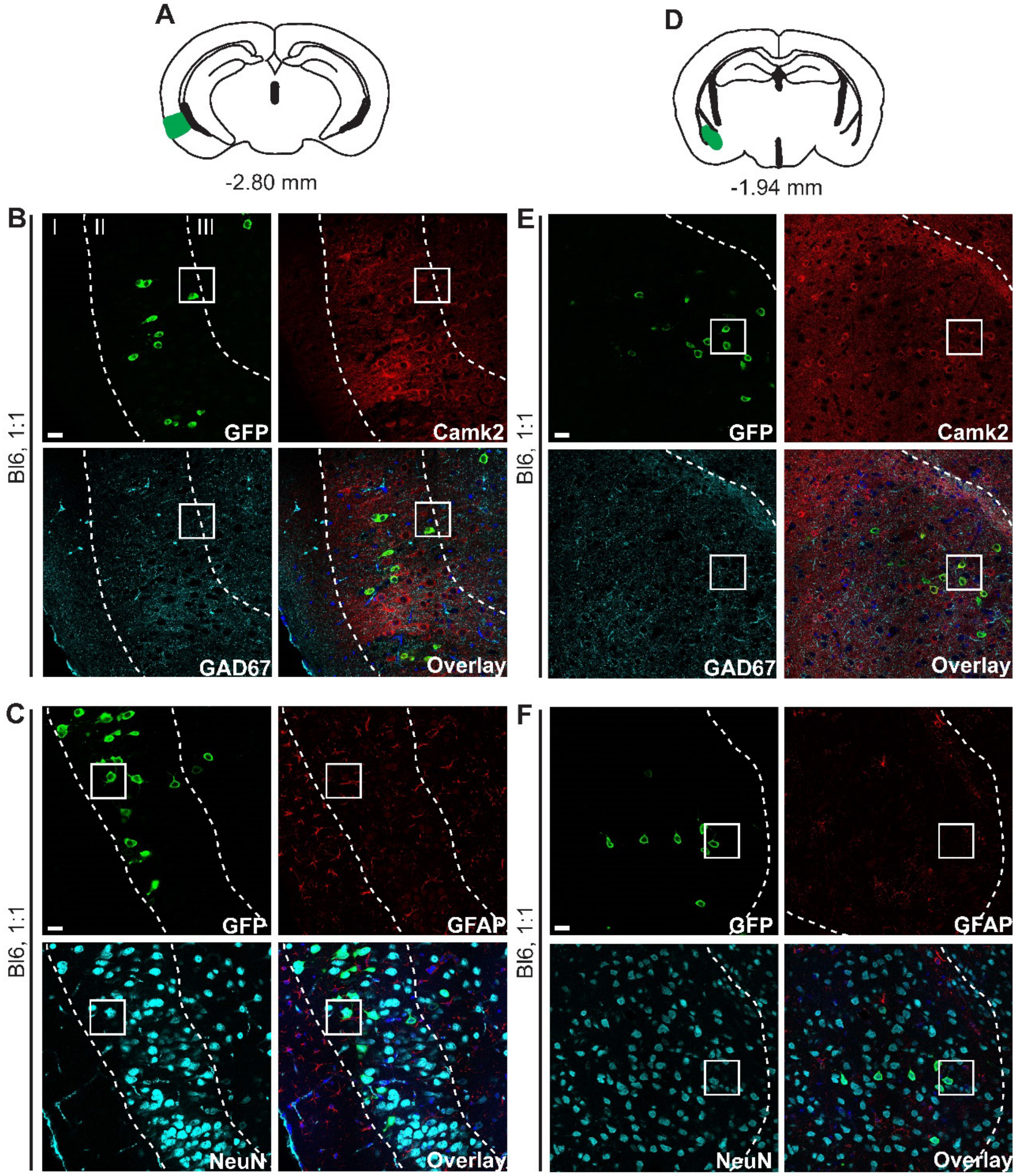

Supplementary Figure 3. Low magnification images of the entorhinal cortex and basal amygdala after injection of AAV9-Camk2a-TRAP in the hippocampus. AAV9-Camk2a-TRAP was injected into the hippocampus of wild type C57BL/6J mice (1:1 viral titer, 2.18 x 10^{13} viral genomes / ml). **(A-C)** Low magnification images of the entorhinal cortex (**A:** highlighted in green) showing immunohistochemical labeling of EGFP-L10a (green) with various cell-type markers (**B:** Camk2a, GAD67; **C**: GFAP, NeuN). **(D-F)** Low magnification images of the basal amygdala (**D**: highlighted in green) showing immunohistochemical labeling of EGFP-L10a (green) with various cell-type markers (**E:** Camk2a, GAD67; **F**: GFAP, NeuN). **(B-C, E-F)** Scale bars 25 μm, filled arrows indicate cells positive for the red channel, open arrows indicate cells positive for the cyan channel, overlay includes DAPI, boxed areas indicate the location of the high magnification images shown in Figure 3.

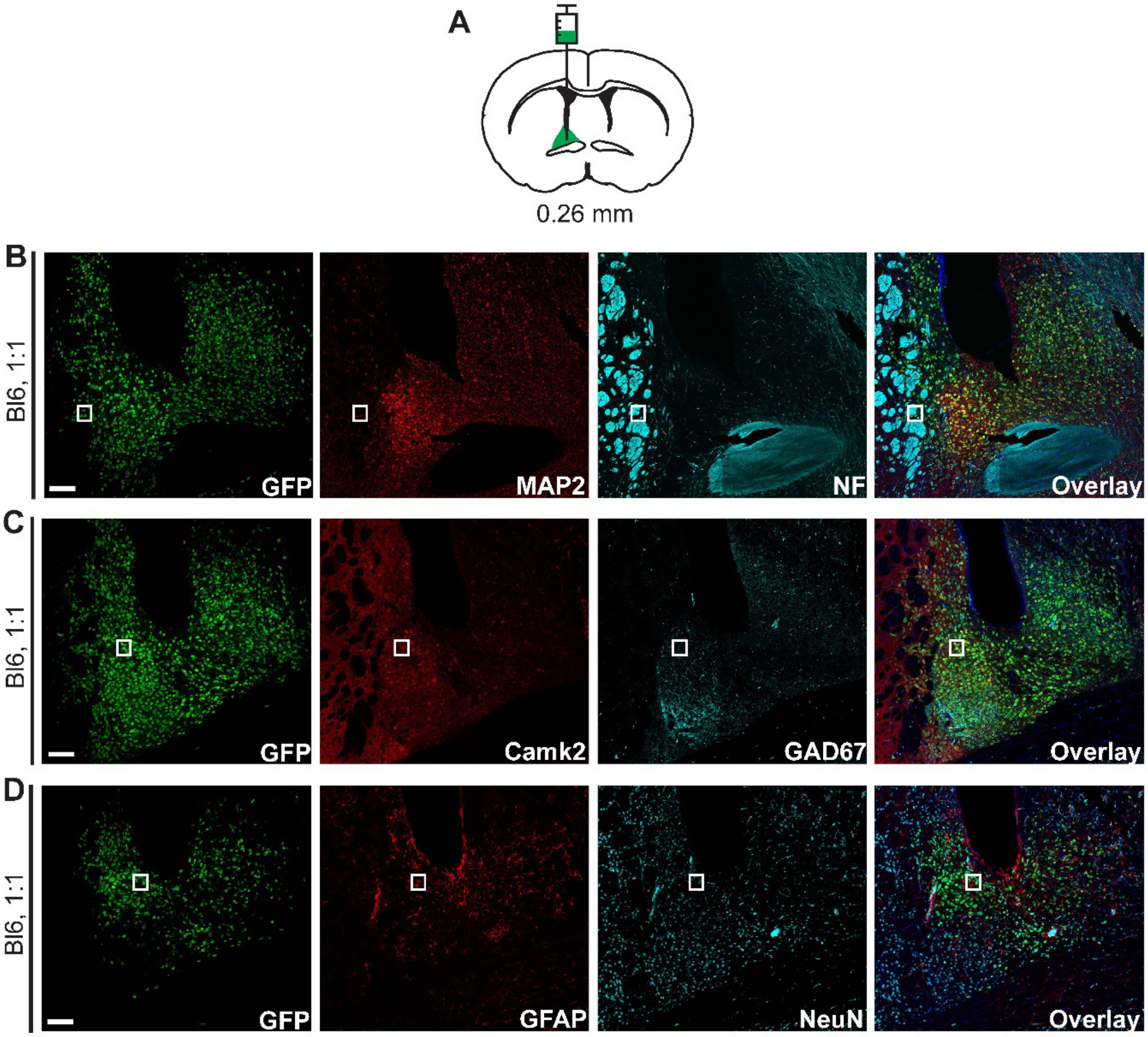

Supplementary Figure 4. Low magnification images of the BNST after injection of AAV9-Camk2a-TRAP in the BNST. (A) AAV9-Camk2a-TRAP was injected into an area containing the anterodorsal, anterolateral, and oval subdivisions of the BNST of wild type C57BL/6J mice (1:1 viral titer, 2.18×10^{13} viral genomes / ml; bregma: 0.26 mm, targeted BNST subdivisions highlighted in green). **(B-D)** Low magnification images of the BNST showing immunohistochemical labeling of EGFP-L10a (green) with various subcellular and cell-type markers (**B**: neuronal somatodendritic marker MAP2, axon marker NF; **C:** Camk2a, GAD67; **D:** GFAP, NeuN). Scale bars 100 μm, overlay includes DAPI, boxed areas indicate the location of the high magnification images shown in Figure 4.

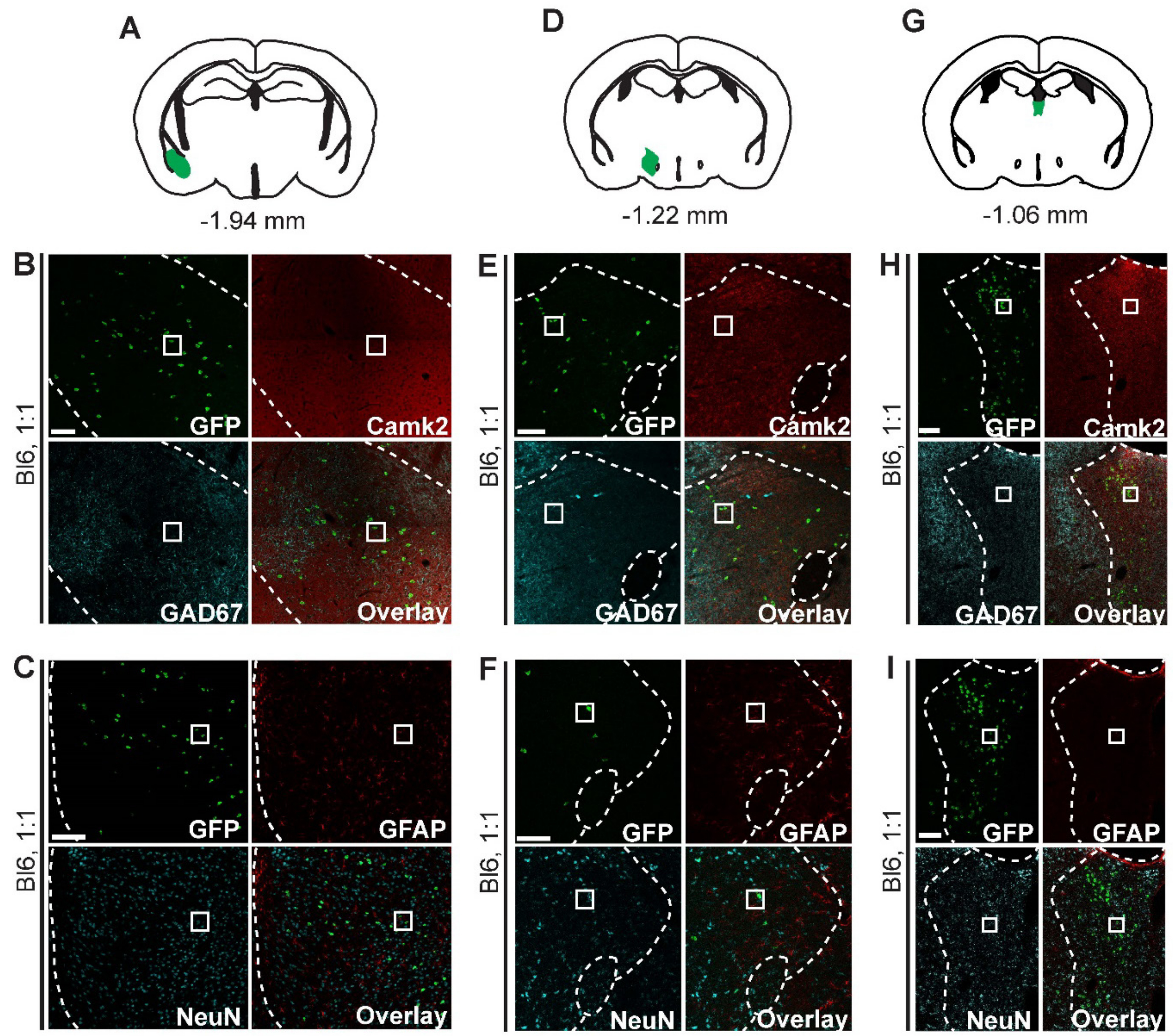

Supplementary Figure 5. Low magnification images of the basal amygdala, lateral hypothalamus, and paraventricular thalamic nucleus after injection of AAV9-Camk2a-TRAP in the BNST. AAV9-Camk2a-TRAP was injected into the BNST of wild type C57BL/6J mice (1:1 viral titer, 2.18 x 10^{13} viral genomes / ml). **(A-C)** Low magnification images of the basal amygdala (**A:** highlighted in green) showing immunohistochemical labeling of EGFP-L10a (green) with various cell-type markers (**B:** Camk2a, GAD67; **C**: GFAP, NeuN). **(D-F)** Low magnification images of the lateral hypothalamus (**D**: highlighted in green) showing immunohistochemical labeling of EGFP-L10a (green) with various cell-type markers (**E:** Camk2a, GAD67; **F**: GFAP, NeuN). **(G-I)** Low magnification images of the paraventricular thalamic nucleus (**G**: highlighted in green) showing immunohistochemical labeling of EGFP-L10a (green) with various cell-type markers (**H:** Camk2a, GAD67; **I**: GFAP, NeuN). **(B-C, E-F, H-I)** Scale bars 100 μm, boxed areas indicate the location of the high magnification images shown in Figure 5.
